# Advanced Medical Education

**Published:** 1893-09

**Authors:** 


BUFFALO MEDICAL AND SURGICAL JOURNAL

A MONTHLY REVIEW OF MEDICINE AND SURGERY.

*EDITORS:*

THOMAS LOTHROP, M. D. - - WM. WARREN POTTER, M. D.

All communications, whether of a literary or business character, should be addressed to the managing editor: 284 FRANKLIN STREET, BUFFALO, N. Y.

VOL. XXXIII. SEPTEMBER, 1893. NO. 2.

### ADVANCED MEDICAL EDUCATION.

THE discussion of the subject of medical education, especially during the last ten years, in and out of the profession, is at last bringing forth its fruits. It is a lamentable fact that we have reason to blush in comparing our standards with those of Europe, or even of Canada. The defects of the system are too plain to be denied. We may plead, in extenuation of these depreciated standards, that the rapid development of our country in its material wealth has resulted in a demand for material in the profession that would meet the exigencies of the times. Standards in medical education, as in other scientific pursuits, have been determined by the direct and practical demands of our people. We are fast approaching the epoch in our national life when the science of medicine here must keep pace with the spirit of research which inspires the profession abroad.

The latest evidence of the growth of this principle in the profession is the organization of the Johns Hopkins Medical School, which opens its first session in October next. We have received the first annual announcement, and also the excellent address of Prof. Welch, defining its objects and its aims. Here the difficulty of fixing the requirements for admission has received the serious consideration the subject demands. "How are we to adapt," writes Prof. Welch, "to the embarrassing and anomalous development of American colleges, a system of medical education for which a liberal education is demanded as a prerequisite ? We are not prepared to recognize a High School training as sufficient, and between this and the training in a college or scientific school there is no intermediate grade. We must, therefore, endeavor to conform to the peculiar conditions in our colleges and scientific schools, by asking that students who are admitted to the medical schools as candidates for the doctor's degree shall possess the liberal education implied by a degree in arts or in science, and shall also have a specified amount of knowledge in certain sciences, as physics, chemistry, and general biology, which are fundamental to the study of medicine."

These words portray the high aims of this new venture in medical education in this country. When taken in connection with the recent advances at Harvard and the prospective advance to a four-years' course at Columbia College in 1894, with the late utterances of the vice-president of the faculty of medicine of Niagara University, who advocates a like advance, it is evident that the long struggle to remedy the deficiencies of the past is at last reaching the end the profession has sought.

The announcement of the Johns Hopkins Medical School furnishes the following requirements for admission: 1. Those who have satisfactorily completed the chemical biological course which leads to the degree of A. B. in that university. 2. Graduates of approved colleges, or scientific schools, who can furnish evidence (*a*) that they have a good reading knowledge of French and German; (*b*) that they have such knowledge of physics, chemistry, and biology as is imparted by the regular minor courses given in these subjects in that university. 3. Those who give evidence, by examination, that they possess the general education implied by a degree in arts or in science from an approved college or scientific school, and the knowledge of French, German, physics, chemistry, and biology already indicated.

These extracts give an idea of the preliminary training required for admission. They are above the standard of any other medical school in this country, and in the world. They indicate the triumph of a principle for which the medical profession has been fighting, and must stimulate other institutions to like efforts in the same direction.

Within two or three years, the Legislatures of New York have established a standard for admission to the medical colleges in this State which amounts to a mere elementary education. The State Board of Medical Examiners also has been established, which furnishes an additional safeguard to the profession. We advocate, in view of the movements already indicated in other States, that this should be raised to the standard of graduation from the High Schools, with sufficient knowledge of Latin and Greek to enable the student to understand the nomenclature of medical terms.

Our State societies should inaugurate a movement in this direction at once. We think the medical schools would cheerfully endorse the principle. The trend of thought in the profession is in that direction, and the movement will go on until the Empire State acquiesces and adopts an advanced standard fully equal to that of the most enlightened States.

